# Multimodal Osteoporosis Assessment using X-ray Imaging and Clinical Data with a Hybrid Deep Learning Model

**DOI:** 10.2174/0115734056488982260824110156

**Published:** 2026-08-31

**Authors:** N.A. Pappathi, B. Sakthivel, Sheik Abdullah S.

**Affiliations:** 1 Department of ECE, NPR College of Engineering and Technology, Dindigul, Anna University -Chennai (Affiliated), Tamil Nadu, India; 2 Department of ECE, Pandian Saraswathi Yadav Engineering College, Sivagangai Dist, Anna University -Chennai (Affiliated), Tamil Nadu, India; 3 School of Electronics, Electrical and Biomedical Technology, Kalasalingam Academy of Research and Education, Krishnankoil Srivilliputhur, Tamil Nadu, India

**Keywords:** Osteoporosis, Dynamic topological convolutional transformer, graph neural networks, Self-organizing maps, Bone Mineral Density, X-ray imaging

## Abstract

**Introduction/Background:**

Osteoporosis is a systemic skeletal disorder characterised by low bone mass. The classification typically includes three stages: normal, osteopenia, and osteoporosis. Early detection is essential for effective treatment and reducing fracture risk. This detection is possible through X-ray imaging and clinical assessments, such as measuring T-score and SoS, etc. This study aims to enhance the accuracy of osteoporosis assessment by combining both imaging and clinical data using advanced AI techniques.

**Materials and Methods:**

A new multimodal approach is proposed for osteoporosis assessment based on imaging and clinical data. The dataset is collected from Begum Shafia Nazir Husain Trust Hospital in Chennai, as well as from various rural clinic camps organized across different regions. For X-ray image classification, a new DL model, the Dynamic Topological Convolutional Transformer (DTCT), is proposed. For clinical data processing, a hybrid model combining Self-Organising Maps (SOM) with Graph Neural Networks (GNN) is applied. The approach is evaluated on a dataset collected from the Begum Shafia Nazir Husain Trust Hospital.

**Results:**

The DTCT-based classification achieves the highest accuracy of 96% for multi-class classification. The SOM-combined GNN model achieves a maximum accuracy of 95% in classification. It achieves a robust framework for integrating different data modalities in medical diagnostics.

**Discussion:**

The proposed method provides high classification accuracy, improved interpretability, robust performance against noise and variations, and a scalable architecture for efficient adaptation to diverse datasets.

**Conclusion:**

The proposed approach combines imaging and clinical data for an accurate osteoporosis assessment with superior performance over existing techniques.

## 1 INTRODUCTION

Osteoporosis is a disease that weakens human bones and makes them much more likely to fracture [1]. Bones are living and rigid connective tissues. It consists of a mineralised matrix of calcium with collagen fibres. Based on age, bones naturally lose some of their density and their ability to regenerate themselves. Osteoporosis can make any of the bones more likely to break. It mostly affects the bones, including knees, hips, wrists, and spine [2, 3]. The most common “symptom” is unexpectedly breaking a bone, especially after a small fall or minor accident. The major symptoms of Osteoporosis are lower back pain and posture change.

A clinician diagnoses osteoporosis with a bone density test. A bone density test uses X-ray imaging to quantify how much calcium and other minerals are in bones [4]. Blood tests may be performed to measure calcium, vitamin D, and certain hormone levels. Recent advances in genomics have shown considerable potential for osteoporosis prediction using genetic variants and polygenic risk scores. However, their routine clinical use remains limited by data availability and cost. Commonly, an X-ray image is used to identify any abnormalities in bone pathologies.

X-ray imagery is the most common imaging procedure amongst the medical community to detect bone pathologies. X-rays can capture images of nearly all the bones in the body, like the wrist, knee, vertebrae, shoulder, hips, and ankles. Likewise, Bone densitometry machines are medical devices that measure Bone Mineral Density (BMD) and assess bone health [5]. These machines are mainly used in diagnosing and managing osteoporosis. It detects low bone density and an increased risk of fractures. Recently, medical imaging technologies have enabled the detection of complex bone disorders, like osteopenia and osteoporosis, with reasonable accuracy [6-9]. A CNN-based approach is proposed by Wani IM *et al*. [10] to find osteoporosis from knee X-rays. They use transfer learning (TL)-based models like AlexNet, VggNet-16, and ResNet for classification. The proposed model is trained and tested on 381 knee X-rays. Compared to all models, the pre-trained AlexNet achieves the highest accuracy of 91.1%. Similarly, Sarhan A.M *et al*. [11] proposed a TL-based CNN model for osteoporosis detection. They applied it to both binary and multi-class classification. Results show that the VGG-19 model reaches a maximum accuracy of 95% when tested on 1,947 knee X-rays.

A classifier model based on a fuzzy broad learning system is proposed by Chen, Z. *et al.* [12] for the detection of osteoporosis in athletes. The texture feature of input images is extracted using the grey-level co-occurrence matrix. Then, a fuzzy broad learning system is used for osteoporosis testing based on extracted features. In their work, M.J.A. Rasool [13] proposed a weighted ensemble technique to differentiate between normal and osteoporotic knee abnormalities. The method is validated through experiments on various CNN-based models with transfer learning. Results showed that the ensemble approach increases accuracy by 4% compared to the individual models.

Naguib, S.M. *et al*. [14] suggested a DL approach based on a superfluity mechanism to classify knee X-ray images. The superfluity mechanism uses two distinct blocks to enable feature flow into two branches instead of a single one. The proposed mechanism is tested on two knee datasets with pre-trained AlexNet and ResNet50. Results show that the Superfluity DL architecture achieves the highest accuracy of 85.42% for dataset1 and 79.39% for dataset2. In their study, L. Dinda *et al.* [15] recommended a multistage approach for osteoporosis detection. Initially, the entire image is divided into regions using a Phi-grid technique. Then, the Vision Transformer is used to extract global features. For classification, the Multi-Layer Perceptron (MLP) classifier is applied. Likewise, Qureshi, M.B. *et al*. [16] proposed a TL-based ResNet-50 for osteoporosis detection. The ResNet-50 model achieves 79% accuracy when tested on 372 X-ray images. Similarly, Wani, I.M. *et al* [17] developed a dataset of 240 knee X-ray images paired with bone mineral density assessments to evaluate osteoporosis. The four different CNN models were used to analyze X-rays. The study found that ResNet and AlexNet achieved the highest accuracy in diagnosing normal and osteoporosis conditions.

Amin, R. *et al*. [18] suggested a customized CNN model for early osteoporosis prediction. For image preprocessing, the bicubic interpolation technique is applied. It highlights an important feature for further processing. The customized model is used for final classification. A hybrid model is suggested by Wang *et al*. [19] for osteoporosis prediction. It combines deep transfer learning (TL) with radiomics features to differentiate abnormalities such as osteoporosis and osteopenia. This feature fusion model achieves higher accuracy and Area Under the Curve (AUC) values for the classification of osteoporosis and osteopenia.

Here, the research gap lies in integrating multi-modal data in a unified framework to effectively capture both spatial image patterns and non-linear relationships in clinical data for more accurate and explainable predictions. To bridge this gap, a multimodal approach is proposed to perform a final classification of bone conditions.

## 2 MATERIALS AND METHODS

### 2.1 Data Collection

In this study, the dataset is collected from Begum Shafia Nazir Husain Trust Hospital in Chennai, as well as from various rural clinic camps organized across different regions. Both X-ray images and clinical data are gathered from all patients. The dataset consists of a total of approximately 9,000 patients, with data collected from both the hospital and rural clinic camps. The data was anonymized and was solely used for academic and research purposes with the certification number IEC/BSH/2025/038. The dataset consists of around 33,000 X-ray images used to classify knee osteoarthritis severity into three categories. Additionally, clinical data is collected from all patients. This clinical data contains key attributes like demographic information, medical history, and physical measurements. The demographic attributes include gender, age, menopause age, height, and weight. The medical history contains details such as diabetes, smoking, alcohol use, previous fractures, family history of osteoporosis, and other relevant health factors. The diagnostic scores include T-score, Z-score, and BMI. The clinical data and X-ray images are carefully labelled and verified by Dr Mohamed Kareem Basha. He is a supervising physician with clinical expertise.

### 2.2 Ethical Considerations

Approval for this study was obtained from the Institutional Ethical Committee of Begum Shafia Nazir Husain Trust Hospital in Chennai (reference number: IEC/BSH/2025/038). All procedures followed in this study were in compliance with the Declaration of Helsinki (1975, updated 2013) and relevant laws and standards. Informed consent was obtained from all participants prior to the collection of data.

### 2.3 Proposed Model

The core of the architecture uses a dual-stream approach: Dynamic Topological Convolutional Transformer (DTCT) and SOM+GNN (Self-Organising Maps and Graph Neural Networks). These two streams are merged in a Fusion stage. The complete steps of the proposed assessment are shown in Fig. (**1**). This combined information leads to a final classification of three stages: Normal, Osteopenia, or Osteoporosis. In the assessment, X-ray imaging provides a complete view of the bone and its loss. Similarly, clinical data like the T-score and Speed of Sound (SoS) measurements give a more complete understanding of a patient's bone health. This combined assessment enhances early detection and improves the precision of risk assessments. The key technical contributions include the following:

(1) **Multi-Scale Fractal Feature Extraction:** The DTCT model incorporates a Fractal Feature Pyramid. It captures fine-grained local and global patterns in X-ray images across multiple scales.

(2) **Fractal Positional Encoding:** Enhances the spatial representation of image patches by injecting hierarchical fractal spatial relationships.

(3) **Topological Self-Attention:** Uses persistent homology-based attention. It is used for the model to focus on topologically significant bone structures.

(4) **Symmetry-Aware Attention:** Uses anatomical symmetry to improve feature consistency across left and right joints.

(5) **Causal Intervention Layer:** Separates causal disease-related features from spurious correlations to enhance generalization.

(6) **Multimodal Fusion Strategy:** Combines image and clinical features using patient-level late fusion with optimized fusion weights (*α*) for improved prediction.

(7) **Robust Training Pipeline:** Uses patient-level dataset splitting and five-fold cross-validation for stable performance assessment.

### 2.4 DTCT

In this work, a novel DL architecture called Dynamic Topological Convolutional Transformer (DTCT) is proposed for multi-class X-ray image classification. DTCT integrates four innovative components for accurate classification: topological self-attention, fractal positional encoding, causal intervention attention, and symmetry-constrained attention.

The architecture uses a multi-head fractal attention mechanism operating simultaneously across different dimensional scales. The topological feed-forward networks process features through persistence-based gating. It is used to process only topologically significant features. The transformer blocks are interleaved with causal disentanglement layers to separate causal from correlative features. The architecture of the DTCT model is shown in Fig. (**2**).

### 2.5 Fractal Feature Pyramid (FFP)

Initially, the input X-ray image is handled by the FFP. It uses multi-scale convolutional layers to capture features at various fractal dimensions. The use of multiple scales allows the model to effectively learn both fine-grained local features and coarser global patterns. The convolutional layers apply different fractal dimensions *d* = 1.3, *d* = 1.5, *d* = 1.8 to capture features at different levels of resolution. Each convolution layer operates at multiple scales. It is used to adapt to different types of image details, which vary in size and importance. The operation can be summarised as:


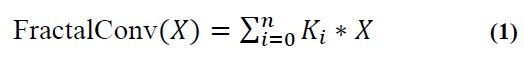


Where *X* is the input feature map, and *K_i_* represents the convolution kernels for each fractal scale. The multi-scale feature extraction is used for the network to understand both micro-level features and macro-level features in the X-ray image. Each branch consists of Conv2D-BatchNorm-ReLU operations with feature aggregation. The first branch uses 64 filters. The second branch uses 128 filters. The third branch uses 256 filters. The Kernel sizes of 3×3 and 5×5 are used to simultaneously capture local texture variations and larger structural bone patterns. The outputs from all branches are concatenated along the channel dimension to form a unified multi-scale representation.

### 2.6 Patch Embedding

After the fractal feature extraction, the image is divided into non-overlapping patches. Then, each patch is processed through a 2D convolutional layer to obtain patch embeddings. This step is used to convert the 2D image into a sequence of patch representations. It can be processed by the transformer model. The patches are flattened into a sequence. It allows the model to handle each patch individually and maintains spatial relationships between them. The embedding process is performed by:


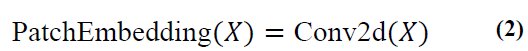


Where the convolution operation extracts features from each patch, and the output is reshaped into a sequence of patch embeddings:


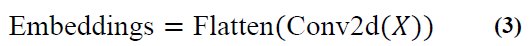


These embeddings are the input for the transformer blocks, where each patch’s embedding carries important information about the image's local features. The feature map is partitioned into 16×16 non-overlapping patches. A convolutional projection layer converts each patch into a 256-dimensional embedding vector. The layer normalization is subsequently applied before passing the embeddings into the transformer encoder.

### 2.7 Fractal Positional Encoding

After patch embeddings, the Fractal Positional Encoding is applied to inject spatial information into the model. Conventional transformers use sinusoidal positional encodings. But the DTCT replaces them with fractal-based positional encodings. These encodings are more flexible and capable of capturing multi-scale spatial patterns. Compared to conventional sinusoidal positional encoding, the proposed fractal positional encoding uses self-similarity information derived from local image structures. The fractal modulation function F(p, i) is computed from local fractal dimension estimates extracted from neighboring image regions. This strategy is used for the model to preserve hierarchical spatial relationships and multi-scale anatomical information. The modulation coefficient is empirically fixed at 0.1 after validation experiments. The positional encoding for a position *p* and embedding dimension *d* is computed as:


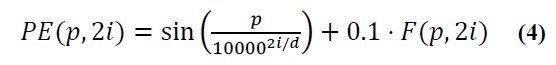



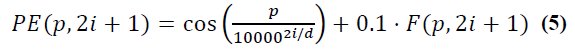


Where *F*(*p*, 2*i*) represents the fractal modulation applied to the positional encoding; this encoding is used for the model to effectively understand the spatial relationships between patches at different levels of detail.

### 2.8 DTCT Blocks (×6)

The core of the DTCT model consists of six DTCT blocks. The transformer layers are designed to process the sequence of patch embeddings. Each block integrates several advanced mechanisms:

### 2.9 Topological Self-Attention

This attention mechanism modulates the standard attention scores by incorporating persistent homology features. These features capture topological structures of holes or connected components. The persistent homology descriptors are extracted from intermediate feature maps using filtration-based topological analysis. The persistence diagrams are transformed into persistence vectors of dimension 128 and subsequently projected into the attention space. The topological term τ(X) is added to the standard self-attention score to emphasize topologically significant structures such as connected trabecular regions and cortical bone boundaries. A persistence threshold of 0.15 is used to suppress topological noise and retain only meaningful anatomical structures. By using topological modulation, the model highlights topologically significant features. The attention score *A* is computed as:


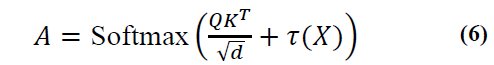


Where,

o *Q = XW_q_* is the query matrix,

o *K = XW_k_* is the query matrix,

o *V = XW_v_* is the query matrix,

o *τ(X)* is the topological modulation term derived from the persistent homology features.

### 2.10 Symmetry-Aware Attention

This mechanism incorporates symmetry embeddings into the attention computation. In medical imaging, symmetry is very important when dealing with symmetric body parts. By appending symmetry embeddings *S* to the keys and values, the model can enforce anatomical consistency in its attention mechanism. To exploit the anatomical symmetry commonly observed in skeletal structures, learnable symmetry embeddings are considered during attention computation. The symmetry embeddings are initialized randomly and optimized jointly with the transformer parameters during training. These embeddings are used for the model to enforce consistency between anatomically corresponding regions and reduce prediction variability caused by imaging artifacts or local noise. The extended keys and values are computed as:


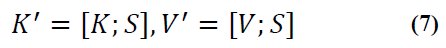


Where S ϵ ℝ^sym_groups×*d*^ represents the symmetry embeddings.

### 2.11 Causal Intervention Layer

This layer uses counterfactual reasoning by applying causal interventions to the feature representations. The model learns how different features contribute to the final prediction and adjusts its representations accordingly to separate causal features from correlative ones. During training, random counterfactual perturbations are introduced through Gaussian noise injection to simulate unseen clinical variations. The intervention parameters μ and σ are initialized using Xavier initialization and updated through backpropagation. This mechanism encourages the model to focus on causal disease-related features rather than dataset-specific correlations. It increases generalization performance across patient populations. The intervention is applied as:


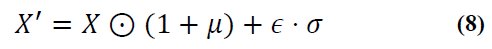


Where *μ* and *σ* are learned intervention parameters, *ϵ* ~ *N* (0,1) is Gaussian noise, simulating counterfactual variations.

### 2.12 Feed-Forward Network

After the attention operations, the feature representations are passed through a feed-forward network. It consists of a linear layer with GELU activation and dropout for regularization. This step refines the features extracted from the attention mechanism. The feed-forward network consists of two fully connected layers with dimensions 256→1024 and 1024→256. The GELU activation is used after the first linear layer with dropout. The residual connections and layer normalization are incorporated after each transformer block to improve training stability and gradient propagation. The feed-forward network is expressed as:


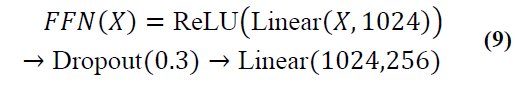


### 2.13 Global Average Pooling

After processing through all transformer blocks, the output sequence is pooled using global average pooling. This operation averages the feature vectors across the sequence dimension. It results in a fixed-length representation of the entire image. The operation is:


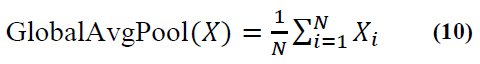


Where *N* is the number of patches, and the result is a vector of fixed size that summarizes the entire image's features.

### 2.14 Classification Head

The pooled feature vector is passed through the classification head. It involves two fully connected layers with ReLU activation and dropout for regularization. This layer reduces the feature vector to the final class probabilities. The classification process is:


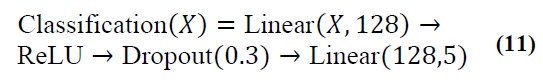


Where *X* is the pooled feature vector, and the final output is a probability distribution over the 5 classes. The final output is a set of class probabilities. It is computed based on the softmax function:


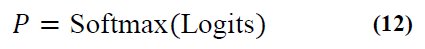


Where the logits are the raw output from the final classification layer, and the softmax function converts them into a probability distribution over the 3 classes.

### 2.15 Hybrid Model for Clinical Data Processing

For clinical osteoporosis classification, in this work, a hybrid Self-Organizing Maps (SOM) based Graph Neural Networks (GNN) [20] is applied. The SOM is used to identify clusters in the data. It represents different patient profiles based on demographic and medical features. Then, a GNN model is trained on the graph to predict osteoporosis and normal diagnoses.

### 2.16 Data Preprocessing

In this stage, the raw dataset is prepared for clustering and classification. This step involves missing value imputation and feature encoding. In Categorical features, missing entries are replaced with the most frequent category. In Numerical features, the missing values in numerical features are imputed with the median value. The categorical variables such as gender, smoking status, and medical histories are encoded using Label Encoding. This process maps each category to a unique integer value. If a categorical variable has *n* distinct categories, it is transformed into integers ranging from 0 to *n* - 1. The label encoding function is:


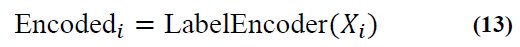


where *X_i_* is the *i*-th categorical feature. The numerical features are standardized to have zero mean and unit variance using Z-score normalization:


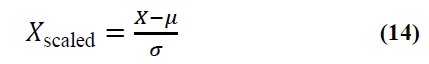


where *µ* is the mean of the feature, and *σ* is the standard deviation.

### 2.17 Clustering with SOM

The SOM is used for unsupervised clustering of the dataset. SOM is trained using a competitive learning algorithm. It converts high-dimensional input data into a lower-dimensional grid of nodes.

The SOM model is trained by applying the following update rule:


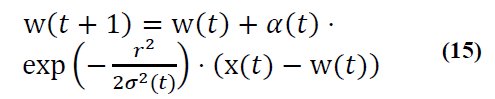


where w(*t*) is the weight vector of the winning neuron at time step *t*, *α*(*t*) is the learning rate, *α*(*t*) is the neighborhood function parameter that decays over time, *r* is the distance between the winning neuron and its neighbors in the grid, and x(*t*) is the input vector at time *t*. Each input sample is assigned to the closest node in the SOM grid, which is determined by the winner-takes-all rule:


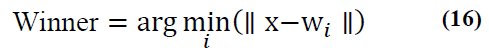


where *w_i_* represents the weight vector of node *i* in the SOM grid. After clustering, a graph structure is created to model the relationships between patients (nodes) and their diagnoses (edges). The nodes represent patients, and the edges represent the similarity between them based on their features. The edges are formed based on k-nearest neighbors (k-NN) within each cluster. The weight of an edge between nodes *i* and *j* is inversely proportional to the distance between them:


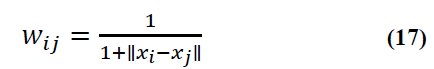


where *w_ij_* the weight of the edge between nodes *i* and *j,* and *x_i_* and *x_j_* are the feature vectors of the nodes.

### 2.18 GNN for Node Classification

The system uses GNN for node classification, where each node is classified into one of the three diagnosis categories: osteopenia, normal, or osteoporosis. The GNN uses graph convolution layers to propagate information through the graph. The Graph Convolution operation aggregates features from neighboring nodes, with the following formula:


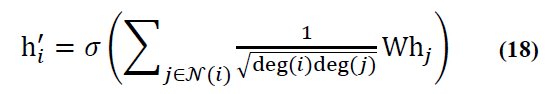


where, h’*_i_* is the updated feature vector for node *i*, *N*(*i*) is the set of neighbors of node *i,* deg(*i*) is the degree of node *i,*Wis the weight matrix, *σ* is the activation function. The final output of the GNN is the predicted class for each node:


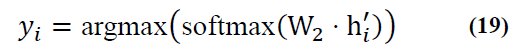


where W_2_ is the output weight matrix, and h’*_i_* is the feature vector for node *i* after graph convolutions.

### 2.19 Fusion Strategy for Multi-Modal Classification

In this work, a Late Fusion strategy is applied to combine the outputs from both image and clinical data classification. This fusion integrates the class probabilities obtained from both modalities at the decision level. The final prediction takes into account both the visual features extracted from the X-ray images and the structured clinical data from the patient profile. Let 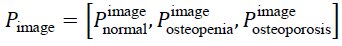 represent the class probabilities from the image classifier, and let 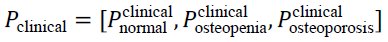 represent the class probabilities from the clinical classifier. To ensure accurate multimodal integration, the outputs generated by the DTCT and SOM+GNN branches are aligned at the patient level using a unique patient identifier. For each patient, the class probability vector obtained from the X-ray image through the DTCT model is paired with the corresponding probability vector generated from the patient’s clinical record using the SOM+GNN model. This alignment is used so that both modalities contribute to the final diagnosis of the same patient and prevent cross-subject information mixing during the fusion process. These probabilities are computed using a softmax function after each classifier processes the input data. For each class *c* and each classifier, the following thresholding rule is applied:


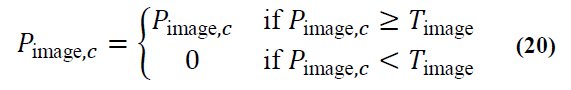



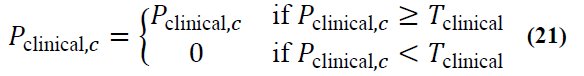


Where, *T*_image_ and *T*_clinical_ are the thresholds for the image and clinical classifiers, respectively. These thresholds can be determined through learning during training. After thresholding, the probabilities from both classifiers are fused to obtain a final probability distribution for each class. A weighted average is used to combine the class probabilities from both modalities. The fused probability for each class *c* is computed as:


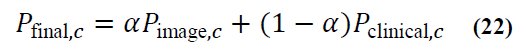


Where *α* is the weight assigned to the image classifier, and (1-*α*) is the weight assigned to the clinical classifier. The fusion weight *α is* optimized via the validation dataset. The candidate values from 0.1 to 0.9 are evaluated. These weights can be tuned based on the relative importance of each modality. The fused probability distribution 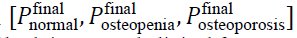 now reflects a combination of both image and clinical features. After probability fusion, a final threshold *T*_final_ is applied to the fused probability distribution. The final fused probability for class *c* is updated as follows:


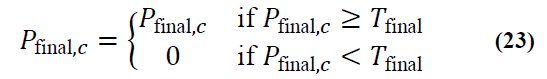


Where *T*_final_ is a global threshold applied to the fused probabilities. If the probability for a class is below the final threshold, it is set to zero. The final prediction is made by selecting the class with the highest fused probability. The predicted class *y*_final_ is determined as:


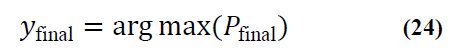


Where, *y*_final_ is the predicted class (Normal, Osteopenia, Osteoporosis), *P*_final_ is the fused probability vector after applying the final threshold.

## 3 RESULTS AND DISCUSSION

The DTCT architecture consists of six transformer blocks with an embedding dimension of 256 and eight attention heads per block. The input X-ray images are resized to 224×224 pixels and normalized to the range [0,^1^]. For preprocessing, histogram equalization and contrast adjustment techniques are applied to increase bone contrast in low-visibility areas. The model is implemented in PyTorch and trained using the AdamW optimizer with a learning rate of 1×10^−4^. The weight decay is set to 1×10^−5^. The batch size is set to 32 with 25 training epochs. The dropout rate is set to 0.3 throughout the network to reduce overfitting. The dataset portioning ratio is given in Table **1**. As this study focused on deep learning model development and validation, conventional statistical power calculation was not applicable. The model reliability is assessed using five-fold cross-validation and external validation. The average of 3 images is collected from each patient with their clinical reports. The demographic characteristics of the study population are given in Table **2**. In testing, the average of 4000 samples is tested for each class. The DTCT model validation plots are given in Fig. (**3**). The training and validation losses both steadily decreased with increased epochs. It is observed that the DTCT generalizes well to unseen images for the classification tasks.

The confusion matrix plot of the DTCT architecture is shown in Fig. (**4**). It shows an exceptionally strong diagonal in classification. It proves its high classification accuracy across all three bone health categories: Normal, Osteopenia, and Osteoporosis. The model correctly identified 3,850 Normal, 3,780 Osteopenia, and 3,900 Osteoporosis cases. Also, the minimal values in the off-diagonal cells demonstrate the model's robustness.

The Receiver Operating Characteristic (ROC) curve of the DTCT model is visualized in Fig. (**5**). The ROC analysis proves that the classification model achieves high diagnostic accuracy across all three categories. The curves indicate that the DTCT is highly effective at deciding between these conditions with a very low rate of false positives (FPR). It maintains a high True Positive Rate (TPR).

The performance of the model for all classes is given in Table **3**. The model shows strong performance across all classes. For the Normal class, it achieves a Precision(Pre) of 0.957, a Recall(Rec) of 0.951, and an F1-score (F1) of 0.954. In the Osteopenia class, the precision is 0.928, the recall is 0.933, and the F1-score is 0.931. The Osteoporosis class has the highest metrics with a precision of 0.962, recall of 0.963, and F1-score of 0.962, respectively.

The evaluation of the DTCT model with existing architectures is given in Table **4**. The proposed DTCT model achieves better performance with an Accuracy(Acc) of 94.90%. It also shows the Pre (0.9490) and Rec (0.94897). The ViT + MLP model shows an accuracy of 92.50%. It has slightly lower Pre (0.9200) and Rec rates(0.9100). The Swin Transformer model reaches an Acc of 90.75%. The Fuzzy ensemble + RF and Pre-trained AlexNet models attain the Acc values of 88.50% and 88.60%, respectively. Overall, the Proposed DTCT model achieves better results in terms of all metrics.

To validate model stability, a 5-fold cross-validation is performed. The model is trained and evaluated five times. Each time, a different fold is used as the test set, and the remaining folds are used for training. The cross-validation results are given in Table **5**. The Proposed DTCT shows the best results in all metrics, with the highest mean accuracy (94.85%) and the most consistent results. The ViT + MLP and Pre-trained ResNet models show slightly more variability. The Fuzzy ensemble + RF and customized CNN models show lower performance, with higher standard deviations. It indicates more inconsistency across folds.

The visualisations of the SHAP Force Plot and SHAP Waterfall are shown in Fig. (**6**). The red bars/arrows represent features that increase the model's output (positive contribution). The blue bars/arrows indicate features that decrease it (negative contribution).

In the “Normal” case, the model reaches a final prediction value of 3.08. The waterfall plot highlights that Feature 17006 (+0.47) and Feature 44609 (+0.37) are the primary drivers increasing the prediction score. For the “Osteopenia” instance, the final prediction value is higher at 3.81. Unlike the normal case, this instance shows a conflicting influence: Feature 40901 acts as a significant “lower” signal (-0.23, shown in blue). The “Osteoporosis” case shows a final prediction value of 3.22. This instance is characterized by a high number of features with moderate positive impacts which led by Feature 50111 (+0.43) and Feature 33688 (+0.37). To improve interpretability, the SHAP feature identifiers are mapped to clinically meaningful variables as given in Table **6**.

Figure **7** shows a Grad-CAM (Gradient-weighted Class Activation Mapping) visualization of the model. In these visualizations, the areas highlighted in red signify the parts of the image that have the highest importance in the model's decision-making process. These areas of interest could correspond to bone structures and abnormalities in the knee. The Grad-CAM technique is used to enhance the interpretability of the model and provide transparency into how the model analyses bone conditions.

In SOM+GNN, the Self-Organizing Map is configured with a 10×10 hexagonal grid of 100 nodes. The initial learning rate α_0_ is set to 0.5. The neighborhood radius σ_0_ is set to 5.0. The training epochs are set to 150. For the GNN, the graph is constructed using k=5 nearest neighbors per node within each SOM cluster. The GNN has 2 graph convolution layers with a hidden dimension of 64. The dropout rate is set to 0.3. All random seeds are set to 42 for reproducibility. For preprocessing, the categorical features are label-encoded. The Numerical features are standardised using Z-score normalization. The missing values are obtained using an imputation technique. Then, the pre-processed clinical feature vectors are mapped using a SOM and subsequently processed by the GNN to capture relationships among features. The training and validation plot of SOM+GNN is shown in Fig. (**8**). The plot shows the training and validation loss plots over the epochs of the model training process. This curve represents the loss calculated on the training data at each epoch. As the model is trained, the training loss decreases steadily. It shows that the model is learning from the training data and increasing its performance over time. Overall, the model is increasing its performance on the training data.

Figure **9** visualises the graph structure colors by diagnosis. The blue nodes represent normal individuals and the red nodes represent those with osteoporosis. The grey lines (edges) connecting the nodes illustrate the similarity between different data points as calculated by the GNN. The model identifies complex patterns in clinical data to distinguish between normal and abnormal bone states.

The feature importance scores for various attributes in a model prediction are shown in Fig. (**10**). The most influential features are the T-score value, SOS value, and Z-score value. These features contribute significantly to the model's predictions. The T-score value has the highest weight for classification. The other important features are age, height, and occupation. These results highlight the key factors that impact the classification outcome.

The SOM-GNN model reaches the highest Acc of 93.83% as given in Table **7**. The Rec and Pre rate of 0.9333 & 0.9586, respectively. The SVM model shows an Acc of 91.50% along with Pre and Rec values of 0.9450 and 0.9125, respectively. The DT model shows the lowest performance with Acc at 89.10%, Pre of 0.9100, and Rec of 0.8750. The F1-score is 0.8922. The LSTM model achieves 92.00% Acc with a Pre of 0.9400 and a Rec of 0.9200, respectively. The CNN+LSTM hybrid model shows 91.50% Acc, with a Pre of 0.9350 and a Rec of 0.9100%. The RF model has 92.30% Acc, with Pre and Rec values of 0.9380 and 0.9300, respectively. Finally, the XGBoost model performs with 91.80% Acc, a Pre of 0.9300, and a Rec of 0.9200, respectively. Overall, the SOM-GNN model outperforms all other models with higher Pre, Rec, and F1-score rates.

The 5-fold cross-validation results are given in Table **8**. The SOM-GNN shows the best overall performance with 93.6% accuracy, and its F1-score (0.9540) suggests a good balance between precision and recall. It is the most reliable model based on these metrics with low variability. It indicates consistent performance across different folds.

The response of the proposed fusion is assessed on a patient dataset. The model achieved an overall accuracy of 93% which proves its ability to classify patients into the three categories of bone health. The results of randomly selecting a subset of 6 patient cases are given in Table **9**.

To validate the model for information leakage analysis, ablation studies are conducted without the T-score and the Z-score. The results are given in Table **10**.

The relatively small performance reduction shows that the SOM-GNN model learns meaningful predictive patterns from additional clinical variables beyond T-score and Z-score alone. To evaluate the generalization capability of the proposed model, an external validation experiment is conducted using the publicly available Mendeley Osteoporosis Dataset (https://data.mendeley.com/datasets/fxjm8fb6mw/2). The dataset contains knee X-ray images and associated clinical risk factors. The validation results are given in Table **11**. The proposed approach achieves an accuracy of 96.14%, precision of 95.96%, recall of 95.81%, F1-score of 95.88%, and AUC of 98.03%. The results prove that the proposed multimodal strategy maintains high performance across datasets collected from different populations and acquisition settings. It proves the strong robustness and generalization capability for osteoporosis screening applications.

## 4 STUDY LIMITATIONS

The DTCT model shows excellent performance with a high classification accuracy across bone health conditions. The training and validation curves demonstrate steady improvement. The confusion matrix and ROC analysis confirm its strong ability to correctly classify instances with minimal errors. Cross-validation results indicate the model’s stability with a mean accuracy of 94.85%. The SOM-GNN model also achieves strong results with 93.83% accuracy. The proposed method provides high classification accuracy, improved interpretability, robust performance against noise and variations, and a scalable architecture for efficient adaptation to diverse datasets. One limitation of the current model is its complexity. It may limit its deployment in resource-constrained environments. To address this, future efforts should focus on optimizing the model for better computational efficiency.

## CONCLUSION

In this work, a new approach for Osteoporosis assessment is proposed. This approach combines both imaging and clinical data processing for the accurate assessment of Osteoporosis. The evaluation was conducted on the dataset from the BEGUM SHAFIA NAZIR HUSAIN TRUST HOSPITAL. The DTCT-based classification achieves the highest accuracy of 96% for multi-class classification. The SOM-combined GNN model achieves a maximum accuracy of 95% in classification. It achieves a robust framework for integrating different data modalities in medical diagnostics. Future work could focus on further improving the model's generalization across different populations and expanding its applicability to other skeletal disorders.

## Figures and Tables

**Fig. (1):**
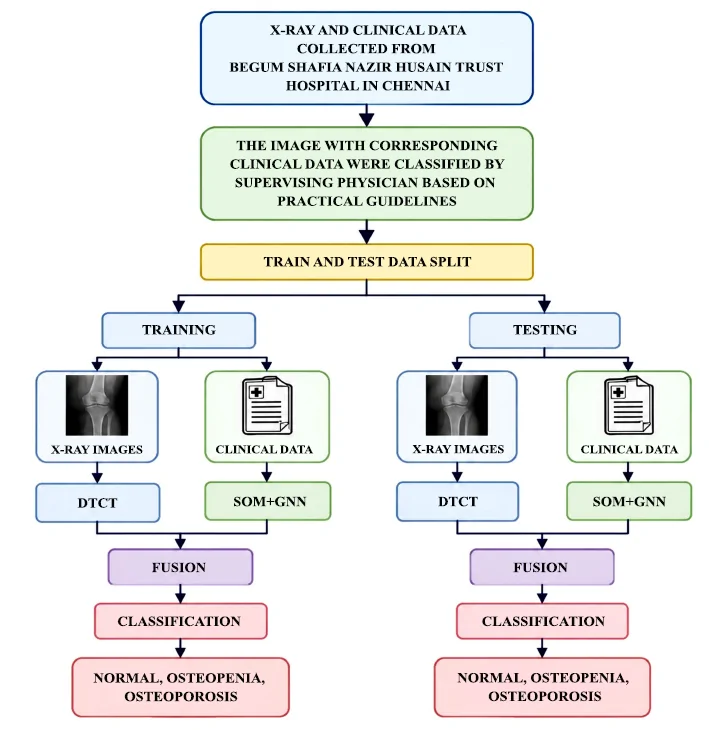
Overall workflow of the proposed osteoporosis assessment.

**Fig. (2):**
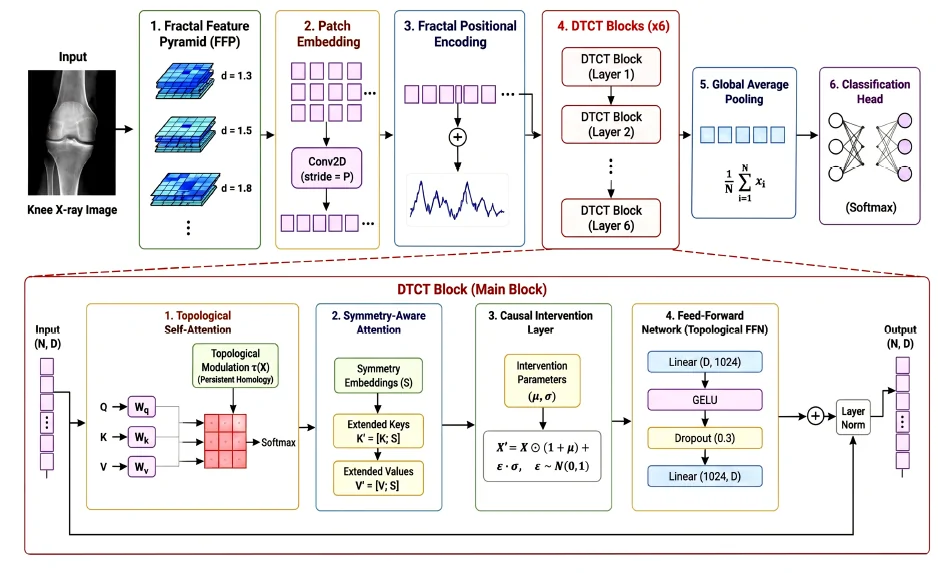
DTCT architecture.

**Fig. (3):**
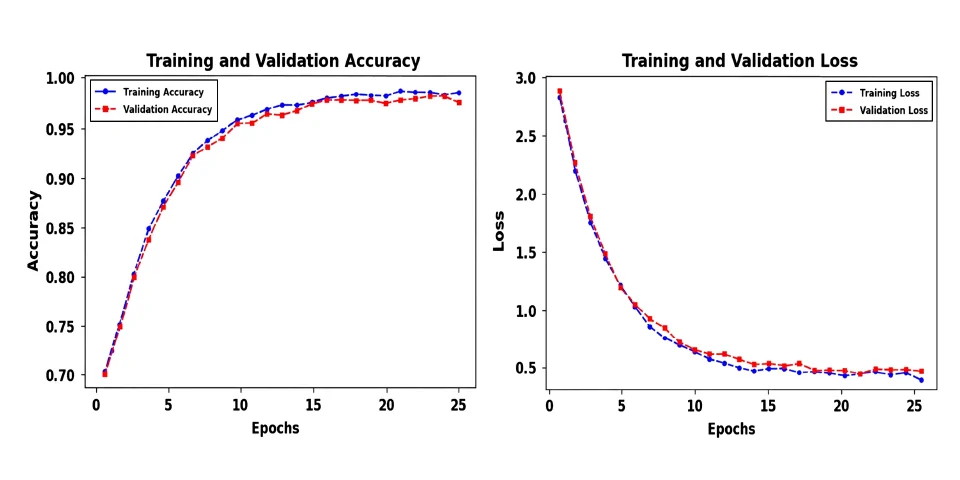
DTCT model validation.

**Fig. (4):**
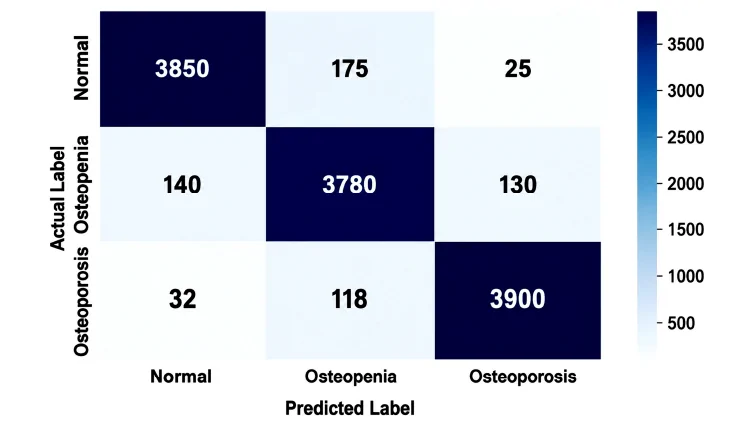
Confusion matrix analysis.

**Fig. (5):**
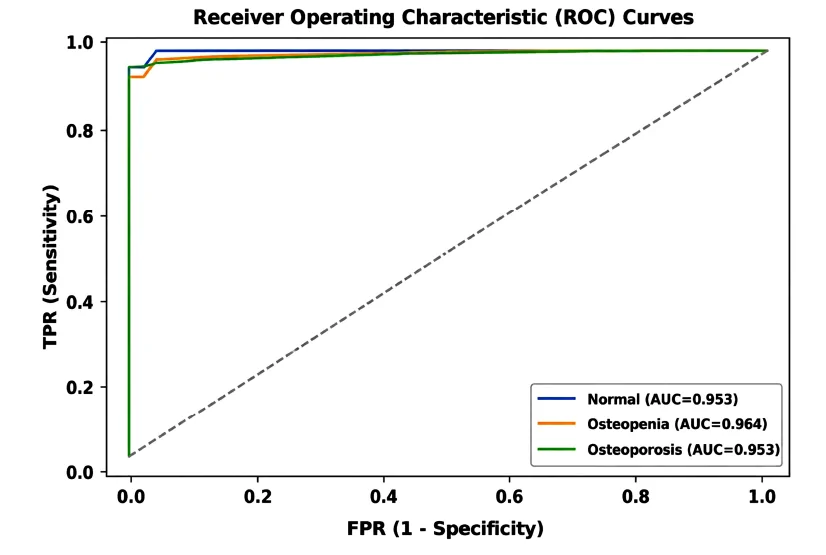
ROC analysis.

**Fig. (6):**
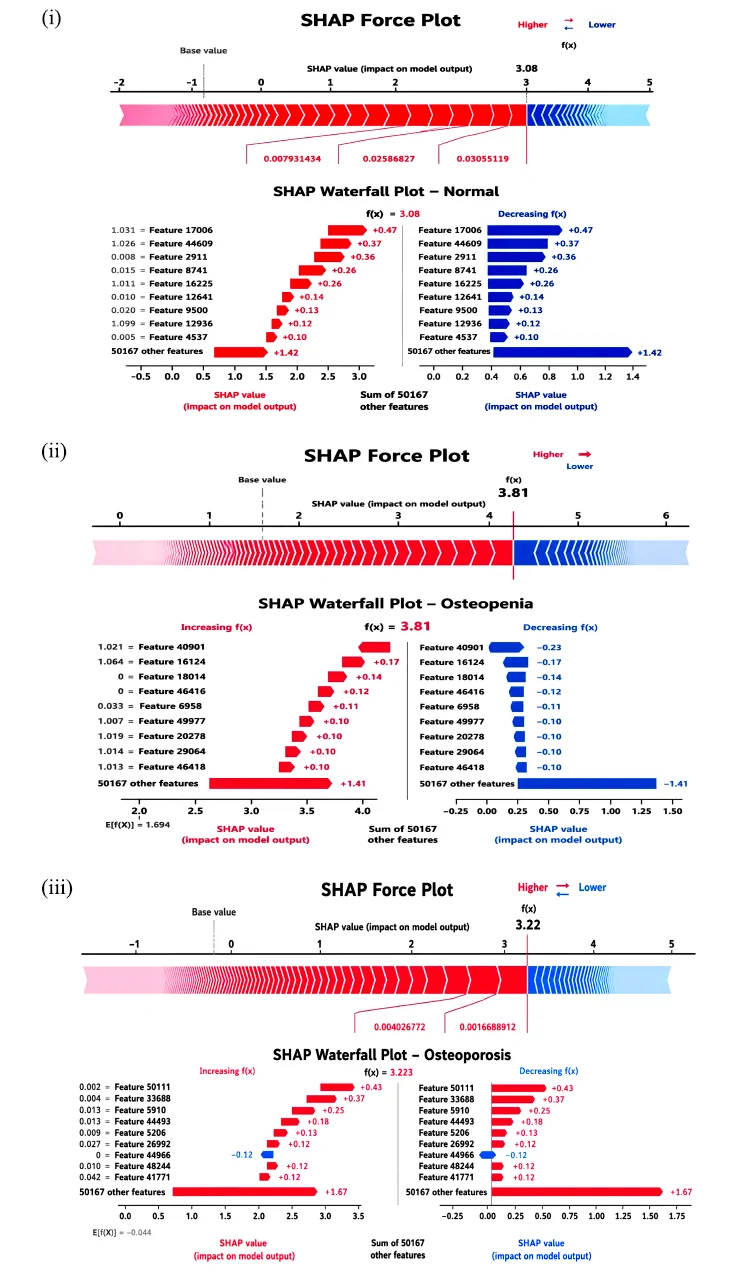
SHAP plots i) normal ii) osteopenia iii) osteoporosis.

**Fig. (7):**
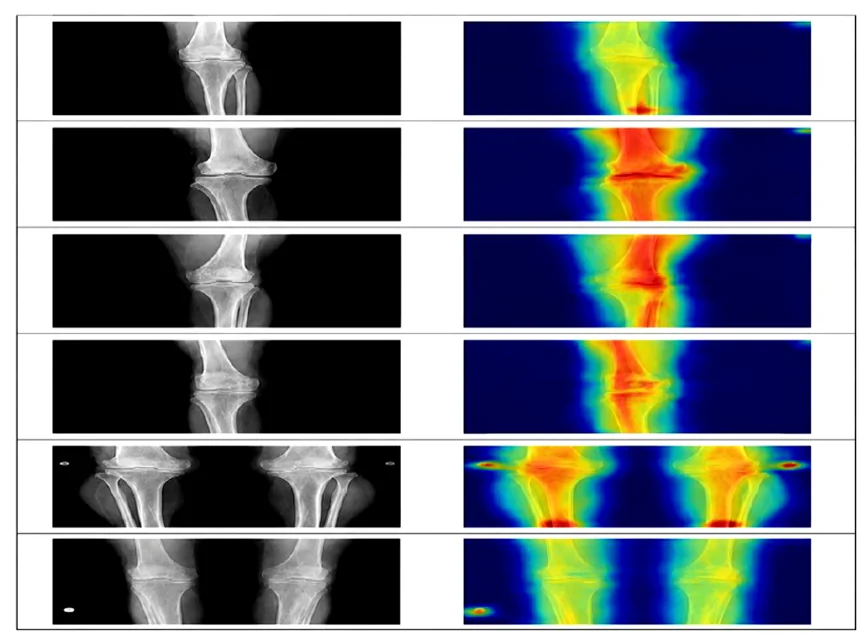
Grad-CAM visualization.

**Fig. (8):**
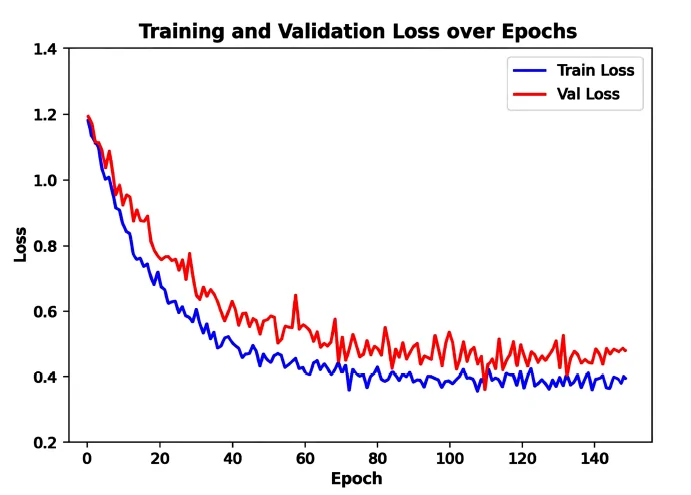
SOM+GNN model validation.

**Fig. (9):**
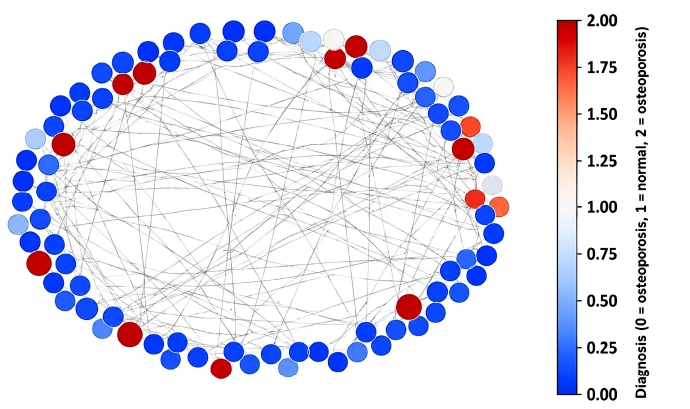
Graph structure colours by diagnosis.

**Fig. (10):**
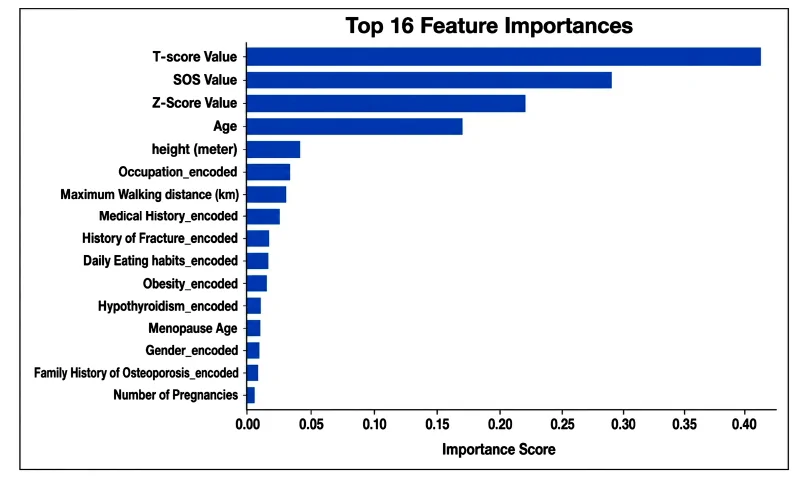
Feature importance plot of clinical data.

**Table 1:** Dataset partitioning.

**Dataset Subset**	**Number of Patients**	**Number of Images**
Training	5,400	18,000
Validation	1,200	3,000
Testing	2,400	12,000
Total	9,000	33,000

**Table 2:** Demographic characteristics of the study population.

Characteristics	Overall (n = 9000)	Normal (n = 3150)	Osteopenia (n = 2900)	Osteoporosis (n = 2950)
Number of patients	9000 (100%)	3150 (35.00%)	2900 (32.22%)	2950 (32.78%)
Age (years), Mean ± SD	59.7 ± 10.8	51.2 ± 8.9	59.8 ± 8.2	68.1 ± 8.7
Gender, n (%)				
Male	3825 (42.50%)	1805 (57.30%)	1189 (41.00%)	831 (28.17%)
Female	5175 (57.50%)	1345 (42.70%)	1711 (59.00%)	2119 (71.83%)
Menopause age (years), Mean ± SD	48.0 ± 4.1	48.4 ± 3.7	48.1 ± 4.0	47.6 ± 4.4
Height (cm), Mean ± SD	158.6 ± 8.7	160.8 ± 8.1	158.4 ± 8.4	156.2 ± 8.9
Weight (kg), Mean ± SD	62.1 ± 11.5	66.1 ± 10.6	61.8 ± 10.8	57.9 ± 11.1
BMI (kg/m^2^), Mean ± SD	24.7 ± 4.2	25.7 ± 3.9	24.6 ± 4.0	23.4 ± 4.1
T-score, Mean ± SD	-1.91 ± 1.16	-0.48 ± 0.41	-1.76 ± 0.36	-3.25 ± 0.58
Z-score, Mean ± SD	-0.96 ± 0.84	-0.31 ± 0.40	-0.88 ± 0.53	-1.63 ± 0.72

**Table 3:** Class-wise performance of the model.

Class	Pre	Rec	F1
Normal	0.95724	0.95062	0.95354
Osteopenia	0.92806	0.93333	0.93068
Osteoporosis	0.96178	0.96296	0.96237

**Table 4:** Performance evaluation of DTCT.

Model	Acc	Pre	Rec	F1-Score
Proposed DTCT	94.90%	0.9490	0.94897	0.94886
ViT + MLP	92.50%	0.9200	0.9100	0.9150
Pre-trained ResNet	91.20%	0.9150	0.9100	0.9125
Swin Transformer	90.75%	0.8850	0.8800	0.8825
Fuzzy ensemble + RF	88.50%	0.8700	0.8600	0.8650
Pre-trained AlexNet	88.60%	0.8750	0.8700	0.8725
Customized CNN model	87.50%	0.8700	0.8600	0.8650

**Table 5:** Performance comparison of the model with other models.

Model	CV Accuracy (mean ± std)	CV Precision (mean ± std)	CV Recall (mean ± std)	CV F1-Score (mean ± std)
Proposed DTCT	94.85% ± 0.30	0.9485 ± 0.010	0.9480 ± 0.015	0.9482 ± 0.012
ViT + MLP	92.40% ± 0.45	0.9200 ± 0.020	0.9100 ± 0.025	0.9145 ± 0.022
Pre-trained ResNet	91.10% ± 0.40	0.9150 ± 0.015	0.9100 ± 0.020	0.9120 ± 0.018
Swin Transformer	90.60% ± 0.50	0.8850 ± 0.030	0.8800 ± 0.035	0.8815 ± 0.033
Fuzzy ensemble + RF	88.40% ± 0.70	0.8700 ± 0.035	0.8600 ± 0.040	0.8645 ± 0.037
Pre-trained AlexNet	88.50% ± 0.65	0.8750 ± 0.030	0.8700 ± 0.035	0.8720 ± 0.033
Customized CNN model	87.40% ± 0.75	0.8700 ± 0.040	0.8600 ± 0.045	0.8645 ± 0.042

**Table 6:** Image feature interpretation.

Feature ID	Anatomical Region
Feature 17006	Femoral neck region
Feature 33688	Proximal tibia
Feature 40901	Cortical bone margin
Feature 44609	Trabecular bone area
Feature 50111	Femoral head region

**Table 7:** SOM-GNN model performance.

**Model**	**Acc**	**Pre**	**Rec**	**F1**
SOM-GNN	93.83%	0.9586	0.9333	0.9558
SVM	91.50%	0.9450	0.9125	0.9285
Decision Tree (DT)	89.10%	0.9100	0.8750	0.8922
LSTM	92.00%	0.9400	0.9200	0.9299
CNN+LSTM	91.50%	0.9350	0.9100	0.9224
Random Forest (RF)	92.30%	0.9380	0.9300	0.9321
XGBoost	91.80%	0.9300	0.9200	0.9250

**Table 8:** SOM-GNN model comparison.

**Model**	**CV Accuracy (mean ± std)**	**CV Precision (mean ± std)**	**CV Recall (mean ± std)**	**CV F1-Score (mean ± std)**
SOM-GNN	93.6% ± 0.04%	0.9570 ± 0.0012	0.9310 ± 0.0052	0.9540 ± 0.0021
SVM	90.5% ± 0.03%	0.9445 ± 0.0020	0.9115 ± 0.0043	0.9260 ± 0.0019
Decision tree	87.2% ± 0.05%	0.9090 ± 0.0032	0.8740 ± 0.0060	0.8915 ± 0.0041
LSTM	91.2% ± 0.04%	0.9390 ± 0.0017	0.9180 ± 0.0040	0.9290 ± 0.0023
CNN+LSTM	89.8% ± 0.05%	0.9330 ± 0.0025	0.9075 ± 0.0045	0.9205 ± 0.0032
Random forest	92.0% ± 0.03%	0.9370 ± 0.0019	0.9290 ± 0.0035	0.9310 ± 0.0020
XGBoost	90.2% ± 0.02%	0.9300 ± 0.0015	0.9190 ± 0.0040	0.9245 ± 0.0025

**Table 9:** Model performance on randomly selected patients.

**Patient ID**	**Predicted Label**	**Fusion Probabilities (Normal, Osteopenia, Osteoporosis)**
0001	Normal	(0.92, 0.04, 0.04)
0512	Osteopenia	(0.04, 0.90, 0.06)
0600	Osteoporosis	(0.03, 0.04, 0.93)
1120	Osteopenia	(0.88, 0.10, 0.02)
5060	Osteoporosis	(0.03, 0.10, 0.87)
7236	Osteoporosis	(0.05, 0.10, 0.85)

**Table 10:** Ablation study.

**Model Configuration**	**Acc**	**Pre**	**Rec**	**F1**
Full Feature Set	93.83%	0.9586	0.9333	0.9558
Without T-score	92.15%	0.9460	0.9180	0.9318
Without Z-score	92.62%	0.9501	0.9210	0.9353
Without T-score & Z-score	91.24%	0.9370	0.9150	0.9259

**Table 11:** External validation on the mendeley osteoporosis dataset.

**Model**	**Acc**	**Pre**	**Rec**	**F1**	**AUC (%)**
DTCT	96.14	95.96	95.81	95.88	98.03

## Data Availability

The dataset used in this study, including both X-ray images and clinical data, is not publicly available due to privacy and confidentiality concerns. However, the dataset is available from the corresponding author [N.A.P] upon reasonable request, with appropriate ethical and legal permissions.

## LIST OF ABBREVIATIONS

AI: Artificial Intelligence
DL: Deep Learning
ML: Machine Learning
DTCT: Dynamic Topological Convolutional Transformer
SOM: Self-Organizing Maps
GNN: Graph Neural Network
CNN: Convolutional Neural Network
BMD: Bone Mineral Density
X-ray: X-ray Imaging
T-score: Bone Density T-score
Z-score: Bone Density Z-score
BMI: Body Mass Index
SHAP: SHapley Additive exPlanations
Grad-CAM: Gradient-weighted Class Activation Mapping
ROC: Receiver Operating Characteristic
AUC: Area Under the Curve
FFP: Fractal Feature Pyramid
FPE: Fractal Positional Encoding
TSA: Topological Self-Attention
SAA: Symmetry-Aware Attention
FFN: Feed Forward Network
RF: Random Forest
SVM: Support Vector Machine
LSTM: Long Short-Term Memory
ViT: Vision Transformer
MLP: Multi-Layer Perceptron
k-NN: k-Nearest Neighbor
TL: Transfer Learning
F1-score: Harmonic Mean of Precision and Recall
CV: Cross Validation
IEC: Institutional Ethics Committee

## STANDARDS OF REPORTING

TRIPOD guidelines were followed.
